# Rate of non-metastatic solid tumor progression following critical illness: a prospective cohort study of UK Biobank participants

**DOI:** 10.62675/2965-2774.20240018-en

**Published:** 2024-10-31

**Authors:** Kathryn Puxty, Rachel Keith, Joanne McPeake, David Morrison, Martin Shaw

**Affiliations:** 1 University of Glasgow School of Medicine, Dentistry and Nursing Glasgow United Kingdom School of Medicine, Dentistry and Nursing, University of Glasgow - Glasgow, United Kingdom.; 2 University of Cambridge The Healthcare Improvement Studies Institute Cambridge United Kingdom The Healthcare Improvement Studies Institute, University of Cambridge - Cambridge, United Kingdom.; 3 University of Glasgow School of Health and Wellbeing Glasgow United Kingdom School of Health and Wellbeing, University of Glasgow - Glasgow, United Kingdom.

**Keywords:** Critical care, Neoplasms, Neoplasms,second primary, Disease progression, Biological specimen banks, Progression-free survival, UK Biobank

## Abstract

**Objective:**

To determine whether admission to critical care is associated with subsequent disease progression in patients with non-metastatic solid tumors.

**Methods:**

This observational cohort study of UK Biobank participants identified those diagnosed with solid tumors and survived hospitalization. Two cohorts were identified based on critical care admission and new metastatic disease as reported at UK Biobank follow-up visits, or primary or secondary care records were compared. Cox proportional hazards analysis was used to account for potential confounders in the multivariate analysis.

**Results:**

A total of 1,854 solid tumor patients were identified, of whom 453 (24.4%) experienced critical care admission. Unadjusted rates of metastatic disease and death were higher for the critical care cohort with lower progression-free survival. At five years, 25% of the critical care survivors and 14% of the hospitalized survivors had developed metastatic disease (p < 0.001), with a corresponding progression-free survival rate of 65% *versus* 81% (p < 0.001). After adjustment for confounders, the hazard ratio for progression-free survival between critical care survivors and the hospitalized cohort was 1.69 (95%CIs 1.31 - 2.18; p < 0.001).

**Conclusion:**

Solid tumor patients admitted to the hospital within 2 years of diagnosis had poorer subsequent progression-free survival if they had experienced a critical care admission. This observation was maintained after adjustment for confounding variables.

## INTRODUCTION

One in twenty patients with a solid tumor is admitted to a intensive care unit (ICU) in the two years following cancer diagnosis.^(1)^ Critical illness can be defined as an acute illness that results in organ dysfunction and is often the result of sepsis, trauma, surgery, or pancreatitis, requiring organ support in the ICU.^(2)^ While the insult leading to critical illness can vary, host immune responses, characterized by hyper- and hypo-inflammatory phases, are common across critical illness etiologies. This dysregulated systemic inflammatory response may have immediate and long-term consequences for cancer patients.

The impact of critical illness on disease progression has not yet been evaluated as a research priority by the clinical community.^(3)^ The mechanism of cancer progression to metastatic disease is poorly understood, but many hypotheses suggest that the host immune response plays a pivotal role in this process.^(4-6)^ The dysregulated immune response during critical illness may modulate the host response to tumor control, with ICU-acquired immunoparesis impacting the tumor microenvironment. Furthermore, ICU survivors often receive a reduction or modification to their anti-cancer therapy in the period following critical illness, with poorer outcomes observed in patients who do not receive treatment.^(7,8)^ All these factors may place cancer patients with critical illness at a greater risk of subsequent metastatic disease.

Our goal was to determine whether admission to critical care is associated with subsequent disease progression in patients with non-metastatic solid tumors.

## METHODS

### Study population

The UK Biobank is a prospective population-based cohort study. Between 2006 and 2010, Biobank recruited over 500,000 participants aged 40 - 70 years from the UK general population. The participants completed baseline health questionnaires, interviews, physical assessments, and sampling. This cohort utilized linked medical records and intermittent further assessment, in-person and online. The external validity of using this cohort to explore exposure-disease relationships has been previously explored, and although the UK Biobank may not be entirely representative of the sample population, using this dataset to explore these relationships is likely to be valid.^(9)^

UK Biobank participants who had a diagnosis of solid tumors (ICD-10 codes C00-80) were identified. Participants were excluded if they had prior documentation of metastatic disease. We identified participants who were admitted to the hospital for more than 24 hours in the two years following their cancer diagnosis. Two study cohorts were then identified from the UK Biobank solid tumor dataset. The primary cohort included those who had survived ICU admission until 30 days post-hospital discharge.

In the United Kingdom, ICU-level care is provided to patients with multiorgan failure or single-organ respiratory failure who need advanced respiratory support. The nurse-patient ratio is 1:1. High dependency care is available to patients with single-organ failure or those requiring high-intensity observation where the nurse-to-patient ratio is approximately 1:2. Beyond professional guidelines, there are no absolute criteria for admission to a critical care area. The final decision rests with the admitting clinician. Generally, patients who have a terminal illness, refuse admission, or have a do-not-resuscitate order are not admitted.

Critical care was defined by consultant specialty at hospitalization (critical or intensive care) within the UK Biobank dataset as described previously.^(10)^ The comparison cohort consisted of patients who survived hospital admission without exposure to ICU 30 days post-discharge. Participants who died within 30 days following hospital discharge were excluded to avoid the inclusion of patients who were discharged from the hospital to end-of-life care. Participants who had existing metastatic disease were also excluded.

### Variables

The Townsend Deprivation Index was used to classify socioeconomic deprivation.^(11)^ This is a census-based index of material deprivation calculated by the combination of four variables for a geographical area: unemployment (percentage of adults economically active), non-car ownership (percentage of households), non-home ownership (percentage of all households), and household overcrowding. A higher Townsend index score implies a greater degree of deprivation.

Comorbidities were assessed via the Charlson Comorbidity Index. ^(12)^ Ethnicity was recorded and categorized accordingly. The date of cancer diagnosis was recorded as the first date of solid tumor diagnosis. The admission type was selected based on the nature of hospital admission.

Metastatic disease was documented as present or absent through the UK Biobank follow-up data analysis. The date of metastasis was defined as the first assessment that recorded the presence of metastatic disease.

### Outcomes

The primary outcome of interest was progression-free survival. The progression-free survival was a composite outcome of survival without progression to metastatic disease. The secondary outcomes included the individual components of progression-free survival, death, and progression to metastatic disease.

### Statistical analysis

The median and interquartile range (IQR) were used to summarize continuous variables, and a Wilcoxon rank-sum test was used to determine differences between medians. Pearson's chi-square test and 95% confidence intervals (95%CI) were used to compare proportions.

Survival analysis was performed for 10-year survival. Participants with insufficient follow-up periods were censored at the last date of known status. Cumulative incidence and Kaplan-Meier survival curves were plotted to analyze the rates of metastasis, mortality, and progression-free survival, and the statistical significance for the differences between cohorts was determined *via* log-rank tests.

**Figure 1:**
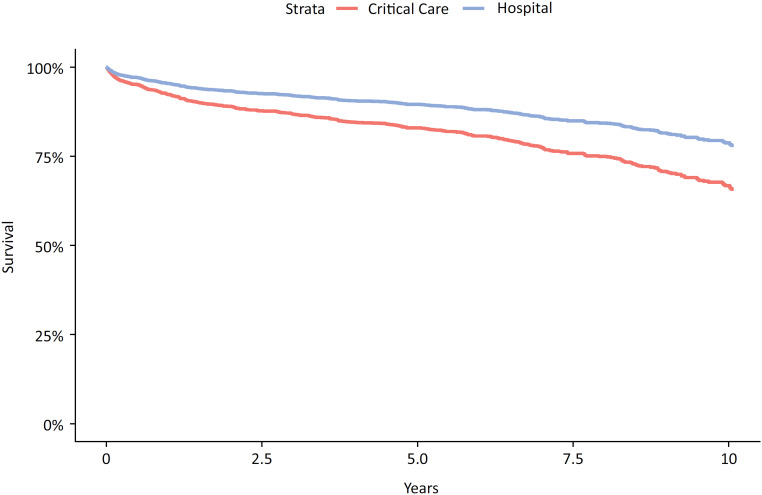
Rate of solid tumor progression (new metastatic disease or death) for patients with non-metastatic solid tumors stratified by critical care (red) or hospital survival (blue). p < 0.001.

Cox proportional hazard models were used to calculate hazard ratios (HR). For the primary outcome, a directed acyclic graph (DAG)^(13)^ was constructed, which included potential causal pathways and mediators for the impact of critical illness on progression-free survival (Figure 1S - Supplementary Material). The variables identified to be included in the multivariable model were age, sex, cancer type, admission type, Charlson score, deprivation index, and smoking status. The proportionality assumption was assessed by visual inspection of survival curves.

R statistical software version 4.1.3 was used to perform computations and analyses.

### Approval

The UK Biobank study received approval from the North-West Multicenter Ethics Research Committee, and all participants provided written informed consent. This study is part of the UK Biobank project 57617 (NHS National Research Ethics Service Ref: 11/NW/0382).

## RESULTS

Among the 502,492 UK Biobank participants, 453 patients had a solid tumor and survived ICU admission, and 1,401 patients had a solid tumor and survived hospitalization without ICU in the two years following cancer diagnosis (Figure 2S - Supplementary Material). Compared with the hospitalized cohort, the critical care cohort was older (median age of 69 years [IQR 65 – 74] *versus* 64 years [IQR 58 – 69]; p < 0.001), with a greater proportion of males (59% *versus* 32%; p < 0.001)) and a higher proportion of patients with multimorbidity (Charlson score ≥ 2 in 57% *versus* 13% (p < 0.001)). The two groups varied in the proportions of different cancers (Table 1S - Supplementary Material). Colorectal cancer (61.2%), bladder cancer (13.5%), esophageal cancer (11.0%), and stomach cancer (8.6%) were the most common cancer types among the critical care cohort. In contrast, the most common cancers in the hospitalized cohort were breast cancer (45.0%), bladder cancer (10.0%), prostate cancer (8.6%) and uterine cancer (8.5%). The nature of hospitalization differed between the groups, with a higher proportion of emergency surgeries and medical admissions in the critical care cohort (17% *versus* 6.1% and 6.1% *versus* 0.9%, respectively).

### Unadjusted outcomes

The unadjusted rates of metastatic disease and death were higher for the critical care cohort, with lower progression-free survival (Figure 3S - Supplementary Material).

At five years, 25% of ICU survivors and 14% of hospitalized survivors had developed metastatic disease (p < 0.001), with a corresponding progression-free survival rate of 65% *versus* 81% (p < 0.001) (Table 1). The unadjusted rate of new metastatic disease or death between ICU *versus* hospital survivors was 20.8% (95%CI 17.1 - 24.6) *versus* 10.7% (95%CI 9.4 - 12.7); p < 0.001 at one year, 35.1% (95%CI 30.7 - 39.5) *versus* 19.1% (95%CI 17.0 - 21.2); p < 0.001 at five years, and 37.5% (95%CI 33.0 - 42.0) *versus* 22.1% (95%CI 19.9 - 24.3); p < 0.001, at ten years.

**Table 1:** Outcomes for critical care survivors and the hospitalized non-critical care cohort

		All patients n = 1,854	Critical care n = 453	Hospital n = 1,401	p value
Progression-free survival (years)					
	1	1,610 (87)	359 (79)	1,251 (89)	< 0.001
	5	1,428 (77)	294 (65)	1,134 (81)	< 0.001
	10	1,374 (74)	283 (62)	1,091 (78)	< 0.001
Mortality (years)					
	1	102 (6)	50 (11)	52 (4)	< 0.001
	5	287 (15)	132 (29)	164 (12)	< 0.001
	10	337 (18)	123 (27)	203 (14)	< 0.001
Metastatic disease (years)					
	1	182 (10)	68 (15)	114 (8)	< 0.001
	5	304 (16)	111 (25)	193 (14)	< 0.001
	10	334 (18)	116 (26)	218 (16)	< 0.001

The results are expressed as n (%).

### Adjusted outcome

After adjustment for the confounders identified (Figure 1S - Supplementary Material), the HR for progression-free survival between critical care survivors and the hospitalized cohort was 1.69 (95%CI 1.31 - 2.18; p < 0.001). Figure 1 shows the adjusted rates between the two cohorts. The corresponding adjusted rate of new metastatic disease or death between ICU survivors and hospital survivors was 11.7% (95%CI 6.9 - 16.2) *versus* 8.6% (95%CI 5.6 - 11.6); p < 0.001 at one year, 23.4% (95%CI 14.4 - 31.3) *versus* 17.6% (95%CI 11.9 - 22.9); p < 0.001 at five years, and 31.1% (95%CI 19.4 - 41.0) *versus* 23.7% (95%CI 16.2 - 30.5); p < 0.001 at ten years.

## DISCUSSION

This analysis revealed an accelerated rate of disease progression in solid tumor patients who survived critical care compared with the hospitalized cohort. One in four critical care survivors and one in seven hospital survivors in the UK Biobank developed new metastatic disease at five years. There was also an increased mortality rate in the ICU survivor group, leading to poorer progression-free survival. The critical care and hospitalized cohorts differed significantly regarding baseline demographics; however, progression-free survival remained poorer for ICU patients after adjustment for the available demographic variables.

These findings, the first to describe rates of disease progression following ICU, have significant consequences for the 5% of solid tumor patients who are admitted to ICU.^(1)^ With increasingly aggressive anti-cancer treatment, we depend on critical care as a facilitator of modern oncological therapies.^(3)^ While increasing survival from critical illness should be celebrated, focusing on longer-term outcomes following discharge is also important.

The UK Biobank has provided a unique opportunity to evaluate long-term outcomes in a large prospective population. The UK Biobank had a response rate of only 5.5% and, consequently, is unlikely to be representative of the general population. As with many self-selected populations, healthy volunteer selection bias occurred, and respondents had a lower cancer incidence than the general population. The participants tended to be from higher socioeconomic groups and may display different health habits. The relatively low rates of lung cancer observed in our populations may reflect this selection bias. However, it remains possible to generalize exposure-disease impacts within the population, such as the effect of ICU admission.^(9)^

Survivorship bias can occur when patients can only be included in a study by first undergoing a selection process. In this study, patients had to survive ICU or hospitalization before inclusion. There may be differences in the characteristics of the patients who survive critical illness compared with those who have died or not encountered critical illness. For example, lung cancer is poorly represented in both cohorts, potentially owing to the high mortality rates observed for these patients.^(1)^ However, as the exposure of interest was ICU, we believe that the inclusion criteria remain the most robust method of testing the hypothesis.

This is an observational study and delineates associations and not causation. While the critical care cohort may have a higher rate of metastatic disease due to the insult sustained during critical illness, it remains likely that the cohorts differ in ways that were not accounted for in our study. Our use of multivariable analysis attempts to overcome those confounders where data are available; however, there may be unmeasured differences between the cohorts which underlined the observed effect. The retrospective nature of this study has limited the data available for analysis, and we could not provide a description of the critical illness, interventions received in ICU, or whether these factors influenced outcomes. A significant weakness of the study is that our dataset does not include contextual data about clinician decision-making about admission decisions, escalation of care, or response to cancer treatment for individual patients.

## CONCLUSION

This study revealed a greater rate of metastatic disease progression in survivors of critical illness; however, the exact cause of this difference cannot be determined, and the impact of specific features of critical illness has not been explored. However, admission practices for intensive care units should not change based on these findings. Instead, we should regard this observation as hypothesis generation, with future studies considering post- intensive care unit progression accounting for the nature and severity of critical illness, degree of immune dysregulation, and subsequent anti-cancer treatment.

## Data Availability

The datasets generated and analyzed during this study are available from the UK Biobank, but restrictions apply to the availability of these data, which were used under license for the current study and are not publicly available. However, data are available *via* application to the UK Biobank.
