# Supplementary material for: Rate of non-metastatic solid tumor progression following critical illness: a prospective cohort study of UK Biobank participants
Source: Crit Care Sci. 2024 Oct 31;36:e20240018en. doi: 10.62675/2965-2774.20240018-en (PMC11634230; doi:10.62675/2965-2774.20240018-en)
Supplement: Supplementary file 1 [file 2965-2774-ccsci-36-e20240018en-Suppl01.pdf]

Critical Care Science
https://doi.org/10.62675/2965-2774.20240018-en

SUPPLEMENTARY MATERIAL

Rate of non-metastatic solid tumor progression following critical illness: a prospective cohort study of UK Biobank participants

Kathryn Puxty[1], Rachel Keith[1], Joanne McPeake[2], David Morrison[3], Martin Shaw[1]

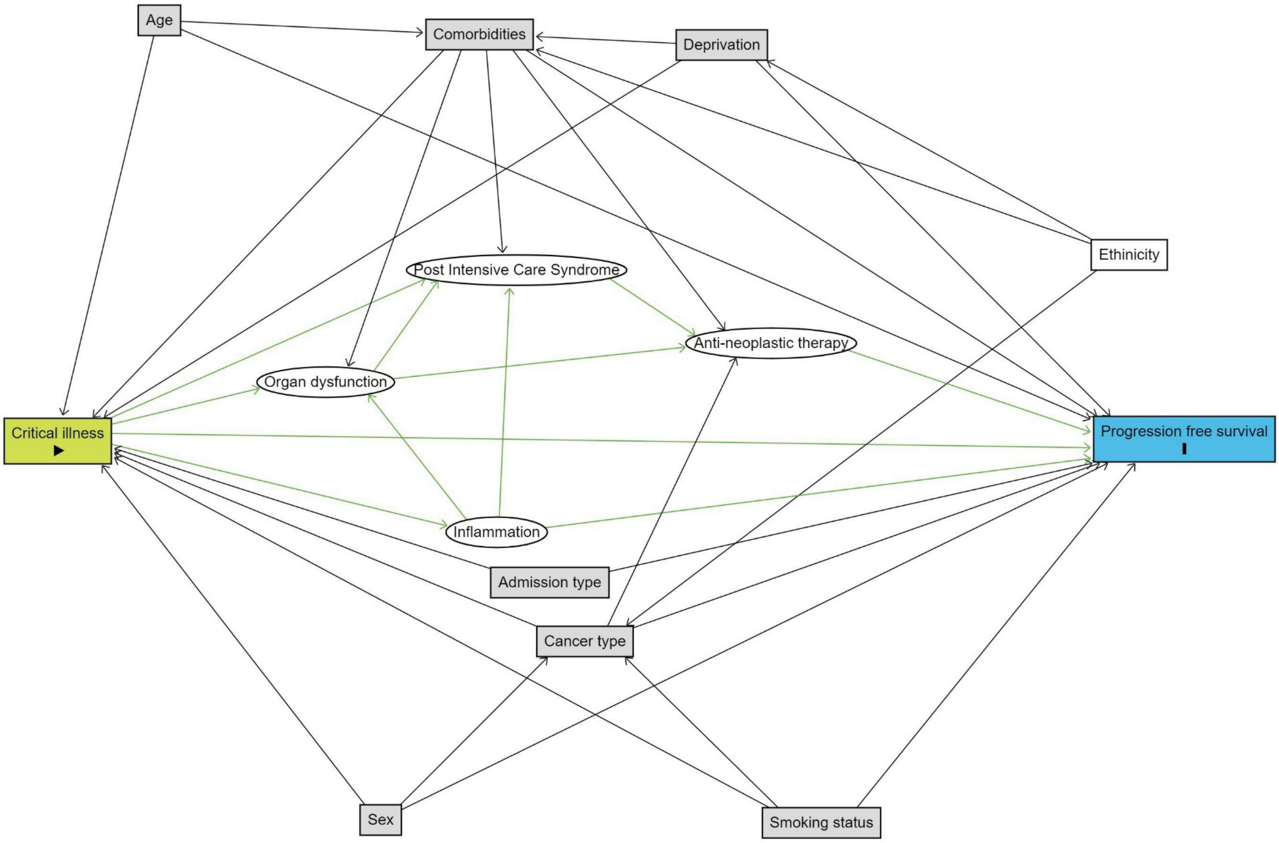

Figure 1S - Directed acyclic graph diagram of potential causal pathways and mediators associated with progression-free survival following Critical illness.

This is an open access article under the CC BY license (https://creativecommons.org/licenses/by/4.0/).

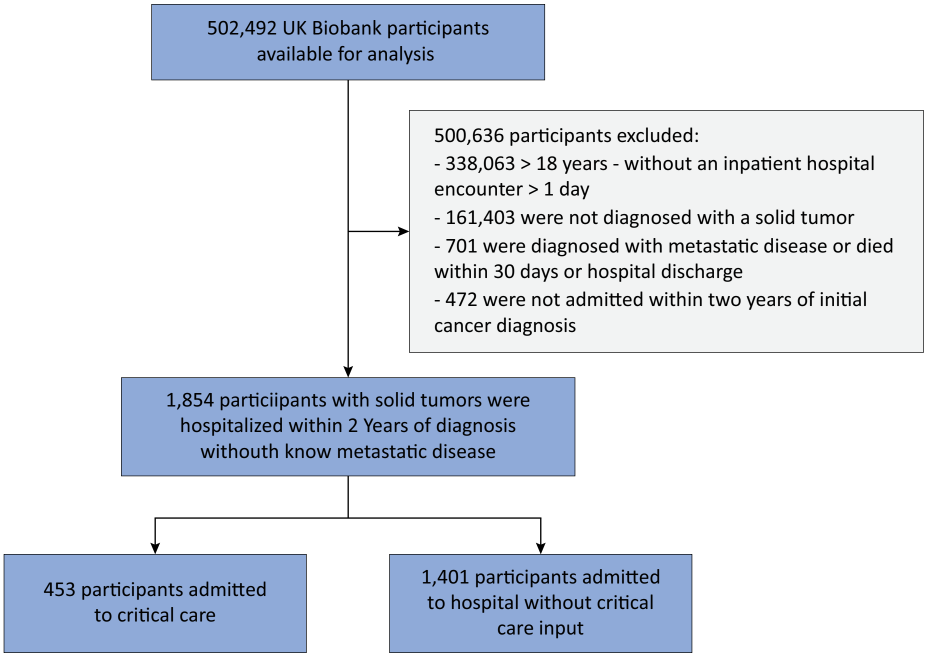

Figure 2S - Consort diagram.

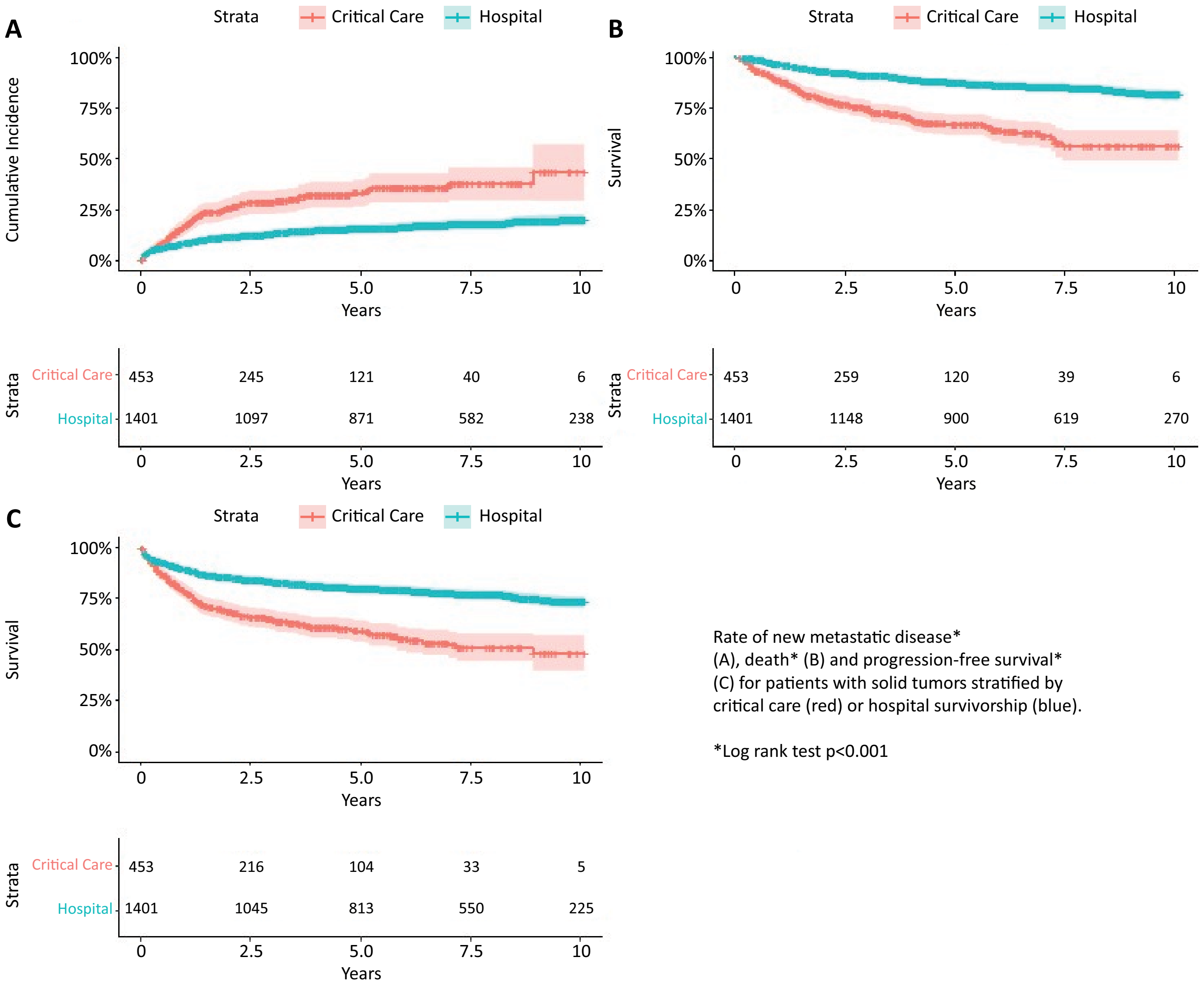

Rate of new metastatic disease*
(A), death* (B) and progression-free survival*
(C) for patients with solid tumors stratified by
critical care (red) or hospital survivorship (blue).

*Log rank test p<0.001

Figure 3S - Long-term outcomes for critical care and hospital survivors.

Table 1S - Demographic features of solid tumor patients stratified by critical care or hospital

Variable	Overall<br>n = 1,854	Critical care<br>n = 453	Hospital<br>n = 1,401	p value
Age at admission	66 (60 - 70)	69 (65 - 74)	64 (58 -69)	< 0.001
Sex				< 0.001
Female	1,142 (62)	185 (41)	957 (68)	
Male	712 (38)	268 (59)	444 (32)	
Deprivation index	-2.40 (-3.76 - 0.12)	-2.48 (-3.68 - 0.57)	-2.38 (-3.78 - 0.02)	0.5
Smoking status				< 0.001
Current	204 (11)	60 (13)	144 (10)	
Never	953 (51)	190 (42)	763 (54)	
Previous	697 (38)	203 (45)	494 (35)	
Ethnicity				0.7
White	1,789 (96)	436 (96)	1,353 (97)	
Other	65 (3.5)	17 (3.8)	48 (3.4)	
Charlson score				< 0.001
0 - 1	1,414 (76)	197 (43)	1,217 (87)	
2+	440 (24)	256 (57)	184 (13)	
Year of cancer diagnosis				< 0.001
< 2006	46 (2.5)	16 (3.5)	30 (2.1)	
2006 - 2010	452 (24)	45 (9.9)	407 (29)	
2011 - 2015	857 (46)	199 (44)	658 (47)	
2016 - 2020	499 (27)	193 (43)	306 (22)	
Malignancy type				< 0.001
Breast cancer	645 (35)	15 (3.3)	630 (45)	
Oral cancer	58 (3.1)	13 (2.9)	45 (3.)	
Oesophageal cancer	56 (3.0)	50 (11.0)	6 (0.4)	
Stomach cancer	40 (2.2)	39 (8.6)	1 (< 0.1)	
Colorectal cancer	206 (11)	126 (61.2)	80 (5.7)	
Lung cancer	59 (3.)	11 (2.4)	48 (3.4)	
Melanoma	45 (2.4%)	0 (0)	45 (3.2)	
Uterine cancer	135 (7.3)	16 (3.5)	119 (8.5)	
Prostate cancer	125 (6.7)	4 (0.9)	121 (8.6)	
Bladder cancer	208 (11)	61 (13.5)	147 (10)	
Other	277 (15)	118 (26.0)	159 (11)	
Admission Year				< 0.001
2006 - 2010	419 (23)	21 (4.6)	398 (28)	
2011 - 2015	871 (47)	193 (43)	678 (48)	
2016 - 2020	564 (30)	239 (53)	325 (23)	
Admission type				< 0.001
Elective surgical	1,660 (90)	357 (79)	1,303 (93)	
Emergency surgical	164 (8.8)	79 (17)	85 (6.1%)	
Medical	30 (1.6)	17 (3.8)	13 (0.)	
Length of stay	2 (1 - 7)	10 (7 - 16)	2 (1 - 3)	< 0.001

Results expressed as n (%) or median (interquartile range).
