# Supplementary material for: Rate of non-metastatic solid tumor progression following critical illness: a prospective cohort study of UK Biobank participants
Source: Crit Care Sci. 2024 Oct 31;36:e20240018en. doi: 10.62675/2965-2774.20240018-en (PMC11634230; doi:10.62675/2965-2774.20240018-en)
Supplement: Translation

# Translation: Taxa de progressão de tumores sólidos não metastáticos após doença crítica: um estudo de coorte prospectivo com participantes do Biobanco do Reino Unido

**DOI:** 10.62675/2965-2774.20240018-pt

**Authors:** Kathryn Puxty, Rachel Keith, Joanne McPeake, David Morrison, Martin Shaw

**Affiliations:** 1 Glasgow Reino Unido School of Medicine, Dentistry and Nursing, University of Glasgow - Glasgow, Reino Unido.; 2 Cambridge Reino Unido The Healthcare Improvement Studies Institute, University of Cambridge - Cambridge, Reino Unido.; 3 Glasgow Reino Unido School of Health and Wellbeing, University of Glasgow - Glasgow, Reino Unido.

**Keywords:** Cuidados críticos, Neoplasias, Segunda neoplasia primária, Progressão da doença, Bancos de espécimes biológicos, Intervalo livre de progressão, Biobanco do Reino Unido

## Abstract

**Objetivo:**

Determinar se a admissão em terapia intensiva está associada à progressão subsequente da doença em pacientes com tumores sólidos não metastáticos.

**Métodos:**

Trata-se de estudo de coorte observacional de participantes do Biobanco do Reino Unido que identificou aqueles diagnosticados com tumores sólidos e que sobreviveram à hospitalização. Duas coortes foram formadas a partir da admissão em terapia intensiva e de uma nova doença metastática, conforme relatado nas consultas de seguimento do Biobanco do Reino Unido, ou foram comparados os registros de Atenção Primária ou Secundária. Utilizou-se a análise de riscos proporcionais de Cox para levar em conta os possíveis fatores de confusão na análise multivariada.

**Resultados:**

Identificaram-se 1.854 pacientes com tumores sólidos, dos quais 453 (24,4%) foram admitidos em terapia intensiva. As taxas não ajustadas de doença metastática e morte foram maiores na coorte de terapia intensiva com menor intervalo livre de progressão. Em 5 anos, 25% dos sobreviventes da terapia intensiva e 14% dos sobreviventes hospitalizados tinham desenvolvido doença metastática (p < 0,001), com taxa de intervalo livre de progressão equivalente de 65% *versus* 81% (p < 0,001). Após o ajuste para fatores de confusão, a razão de risco de intervalo livre de progressão entre os sobreviventes de terapia intensiva e a coorte hospitalizada foi de 1,69 (IC95% 1,31 - 2,18; p < 0,001).

**Conclusão:**

Pacientes com tumores sólidos internados no hospital dentro de 2 anos após o diagnóstico teriam intervalo livre de progressão subsequente mais baixo se tivessem passado por uma internação em terapia intensiva. Essa observação se manteve após o ajuste para variáveis de confusão.

## INTRODUÇÃO

Entre os pacientes com tumores sólidos, um em cada 20 é admitido em uma unidade de terapia intensiva (UTI) nos 2 anos seguintes ao diagnóstico de câncer.^(1)^ Define-se doença crítica como uma doença aguda que resulta em disfunção orgânica e geralmente é resultado de sepse, trauma, cirurgia ou pancreatite, exigindo suporte de órgãos na UTI.^(2)^ Embora o insulto que leva à doença crítica possa variar, as respostas imunes do hospedeiro, caracterizadas por fases hiper e hipoinflamatórias, são comuns em todas as etiologias de doença crítica. Essa resposta inflamatória sistêmica desregulada pode ter consequências imediatas e a longo prazo nos pacientes com câncer.

O impacto da doença crítica na progressão da doença ainda não foi avaliado como uma prioridade de pesquisa pela comunidade clínica.^(3)^ O mecanismo de progressão do câncer até a doença metastática é pouco compreendido, mas muitas hipóteses sugerem que a resposta imune do hospedeiro desempenha papel fundamental nesse processo.^(4-6)^ A resposta imune desregulada durante a doença crítica pode modular a resposta do hospedeiro ao controle do tumor, com a imunoparesia adquirida na UTI afetando o microambiente tumoral. Além disso, os sobreviventes de UTI geralmente têm sua terapia anticâncer reduzida ou modificada no período após a doença crítica, com desfechos menos favoráveis observados em pacientes que não recebem tratamento.^(7,8)^ Todos esses fatores podem colocar os pacientes com câncer e doença crítica em um risco maior de doença metastática subsequente.

Nosso objetivo foi determinar se a admissão em terapia intensiva está associada à progressão subsequente da doença em pacientes com tumores sólidos não metastáticos.

## MÉTODOS

### População do estudo

O Biobanco do Reino Unido consiste em um estudo de coorte prospectivo e populacional. Entre 2006 e 2010, o Biobanco do Reino Unido recrutou mais de 500 mil participantes com idade entre 40 e 70 anos da população geral do Reino Unido. Os participantes responderam a questionários de saúde, entrevistas, avaliações físicas e amostragem iniciais. Essa coorte utilizou registros médicos vinculados e avaliações adicionais intermitentes, presenciais e *on-line*. A validade externa do uso dessa coorte para explorar as relações entre exposição e doença foi explorada anteriormente e, embora o Biobanco do Reino Unido possa não ser totalmente representativo da população da amostra, é provável que o uso desse conjunto de dados para explorar essas relações seja válido.^(9)^

Identificaram-se os participantes do Biobanco do Reino Unido que tinham diagnóstico de tumores sólidos (códigos C00-80 da CID-10). Os participantes foram excluídos se tivessem documentação prévia de doença metastática. Identificamos os participantes que foram admitidos no hospital por mais de 24 horas nos 2 anos seguintes ao diagnóstico de câncer. Duas coortes de estudo foram então identificadas a partir do conjunto de dados de tumores sólidos do Biobanco do Reino Unido. A coorte principal incluiu os participantes que sobreviveram à internação em UTI até 30 dias após a alta hospitalar.

No Reino Unido, a assistência em nível de UTI é fornecida a pacientes com insuficiência de múltiplos órgãos ou insuficiência respiratória de um único órgão que precisem de suporte respiratório avançado. A proporção enfermeiro-paciente é de 1:1. A assistência de alta dependência está disponível para pacientes com insuficiência de um único órgão ou para aqueles que precisam de observação de alta intensidade, sendo a proporção enfermeiro-paciente de aproximadamente 1:2. Além das diretrizes profissionais, não há critérios absolutos para admissão em uma área de UTI. A decisão final cabe ao médico responsável pela admissão. Em geral, não são admitidos pacientes com doença terminal, que recusam a admissão ou que têm uma ordem de não ressuscitar.

Os cuidados críticos foram definidos pela especialidade do consultor na hospitalização (cuidados críticos ou intensivos) no conjunto de dados do Biobanco do Reino Unido, conforme descrito anteriormente.^(10)^ A coorte de comparação consistiu em pacientes que sobreviveram à admissão hospitalar sem exposição à UTI até 30 dias após a alta. Os participantes que evoluíram para óbito dentro de 30 dias após a alta hospitalar foram excluídos para evitar a inclusão de pacientes que receberam alta do hospital para cuidados de fim de vida. Excluíram-se também os participantes que tinham doença metastática.

### Variáveis

O *Townsend Deprivation Index* foi usado para classificar a privação socioeconômica.^(11)^ É um índice de privação material baseado no censo, calculado pela combinação de quatro variáveis de uma área geográfica: desemprego (porcentagem de adultos economicamente ativos), não propriedade de carro (porcentagem de domicílios), não propriedade de casa (porcentagem de todos os domicílios) e superlotação domiciliar. Uma pontuação mais alta no *Townsend Deprivation Index* sugere grau maior de privação.

As comorbidades foram avaliadas pelo Índice de Comorbidade de Charlson.^(12)^ A etnia foi devidamente registrada e categorizada. A data do diagnóstico de câncer foi registrada como a primeira data do diagnóstico de tumor sólido. O tipo de admissão foi selecionado com base na natureza da admissão hospitalar.

A doença metastática foi documentada como presente ou ausente, por meio da análise de dados de seguimento do Biobanco do Reino Unido. A data da metástase foi definida como a primeira avaliação que registrou a presença de doença metastática.

### Desfechos

O desfecho primário de interesse foi o intervalo livre de progressão, o qual, por sua vez, foi um desfecho composto de sobrevida sem progressão para doença metastática. Os desfechos secundários incluíram os componentes individuais de intervalo livre de progressão, morte e progressão para doença metastática.

### Análise estatística

A mediana e o intervalo interquartil (IIQ) foram usados para resumir as variáveis contínuas, e o teste de soma de classificação de Wilcoxon foi usado para determinar as diferenças entre as medianas. O teste do qui-quadrado de Pearson e os intervalos de confiança de 95% (IC95%) foram usados para comparar proporções.

A análise de sobrevida foi realizada para a sobrevida de 10 anos. Os participantes com períodos de seguimento insuficientes foram censurados na última data de condição conhecida. A incidência cumulativa e as curvas de sobrevida de Kaplan-Meier foram traçadas para analisar as taxas de metástase, a mortalidade e o intervalo livre de progressão. A significância estatística das diferenças entre as coortes foi determinada por meio de testes de *log-rank*.

Utilizaram-se modelos de risco proporcional de Cox para calcular as razões de risco (RRs). Para o desfecho primário, criou-se um gráfico acíclico dirigido (DAG - *directed acyclic graph*),^(13)^ que incluía possíveis caminhos causais e mediadores de impacto da doença crítica no intervalo livre de progressão (Figura 1S - Material Suplementar). As variáveis identificadas para serem incluídas no modelo multivariável foram idade, sexo, tipo de câncer, tipo de admissão, Índice de Comorbidade de Charlson, índice de privação e tabagismo. A suposição de proporcionalidade foi avaliada pela inspeção visual das curvas de sobrevida.

Utilizou-se o *software* estatístico R versão 4.1.3 para realizar cálculos e análises.

### Aprovação

O estudo Biobanco do Reino Unido recebeu aprovação do *North-West Multicenter Ethics Research Committee*, e todos os participantes assinaram um Termo de Consentimento Livre e Esclarecido. Este estudo faz parte do projeto UK Biobank 57617 (Serviço Nacional de Ética em Pesquisa do *National Health Service* Ref.: 11/NW/0382).

## RESULTADOS

Dos 502.492 participantes do Biobanco do Reino Unido, 453 pacientes tinham um tumor sólido e sobreviveram à internação em UTI, e 1.401 pacientes tinham um tumor sólido e sobreviveram à hospitalização sem UTI nos 2 anos seguintes ao diagnóstico de câncer (Figura 2S - Material Suplementar). Em comparação com a coorte hospitalizada, a coorte de cuidados intensivos era mais velha (idade mediana de 69 anos [IQ25 - 75% 65 – 74] *versus* 64 anos [IIQ25 - 75% 58 - 69]; p < 0,001), com proporção maior de homens (59% *versus* 32%; p < 0,001) e de pacientes com multimorbidade (escore ≥ 2 do Índice de Comorbidade de Charlson em 57% *versus* 13% (p < 0,001). Os dois grupos variaram nas proporções de cânceres diferentes (Tabela 1S - Material Suplementar). O câncer colorretal (61,2%), o de bexiga (13,5%), o de esôfago (11,0%) e o de estômago (8,6%) foram os tipos mais comuns na coorte de cuidados intensivos. Por outro lado, os cânceres mais comuns na coorte hospitalizada foram de mama (45,0%), de bexiga (10,0%), de próstata (8,6%) e de útero (8,5%). A natureza da hospitalização diferiu entre os grupos, com proporção maior de cirurgias de emergência e internações médicas na coorte de cuidados intensivos (17% *versus* 6,1% e 6,1% *versus* 0,9%, respectivamente).

### Desfechos não ajustados

As taxas não ajustadas de doença metastática e morte foram maiores na coorte cuidados de intensivos, com menor intervalo livre de progressão (Figura 3S - Material Suplementar).

Em 5 anos, 25% dos sobreviventes de UTI e 14% dos sobreviventes hospitalizados tinham desenvolvido doença metastática (p < 0,001), com taxa de intervalo livre de progressão equivalente a 65% *versus* 81% (p < 0,001) (Tabela 1). A taxa não ajustada de nova doença metastática ou morte entre sobreviventes de UTI *versus* de hospital foi de 20,8% (IC95% 17,1 - 24,6) *versus* 10,7% (IC95% 9,4 - 12,7), com p < 0,001 em 1 ano; 35,1% (IC95% 30,7 - 39,5) *versus* 19,1% (IC95% 17,0 - 21,2), com p < 0,001 em 5 anos; e 37,5% (IC95% 33,0 - 42,0) *versus* 22,1% (IC95% 19,9 - 24,3), com p < 0,001 em 10 anos.

**Tabela 1:** Desfechos dos sobreviventes de terapia intensiva e da coorte de pacientes hospitalizados sem cuidados intensivos

		Todos os pacientes n = 1.854	Cuidados intensivos n = 453	Hospitalizados n = 1.401	Valor de p
Intervalo livre de progressão (anos)					
	1	1.610 (87)	359 (79)	1.251 (89)	< 0,001
	5	1.428 (77)	294 (65)	1.134 (81)	< 0,001
	10	1.374 (74)	283 (62)	1.091 (78)	< 0,001
Mortalidade (anos)					
	1	102 (6)	50 (11)	52 (4)	< 0,001
	5	287 (15)	132 (29)	164 (12)	< 0,001
	10	337 (18)	123 (27)	203 (14)	< 0,001
Doença metastática (anos)					
	1	182 (10)	68 (15)	114 (8)	< 0,001
	5	304 (16)	111 (25)	193 (14)	< 0,001
	10	334 (18)	116 (26)	218 (16)	< 0,001

Resultados expressos como n (%).

### Desfecho ajustado

Após o ajuste dos fatores de confusão identificados (Figura 1S - Material Suplementar), a RR do intervalo livre de progressão entre os sobreviventes de UTI e a coorte hospitalizada foi de 1,69 (IC95% 1,31 - 2,18; p < 0,001). A figura 1 mostra as taxas ajustadas das duas coortes. A taxa ajustada correspondente de nova doença metastática ou morte entre os sobreviventes da UTI e os sobreviventes hospitalizados foi de 11,7% (IC95% 6,9 - 16,2) *versus* 8,6% (IC95% 5,6 - 11,6), com p < 0,001 em 1 ano; 23,4% (IC95% 14,4 - 31,3) *versus* 17,6% (IC95% 11,9 - 22,9), com p < 0,001 em 5 anos; e 31,1% (IC95% 19,4 - 41,0) *versus* 23,7% (IC95% 16,2 - 30,5), com p < 0,001 em 10 anos.

**Figura 1:** Taxa de progressão de tumor sólido (nova doença metastática ou morte) em pacientes com tumores sólidos não metastáticos estratificados por sobrevida em cuidados críticos (vermelho) vou hospitalar (azul). p < 0.001.

## DISCUSSÃO

Esta análise revelou taxa acelerada de progressão da doença em pacientes com tumores sólidos que sobreviveram à UTI em comparação com a coorte hospitalizada. Dos sobreviventes de cuidados intensivos, um em cada quatro e um em cada sete sobreviventes hospitalares do Biobanco do Reino Unido desenvolveram nova doença metastática em 5 anos. Observou-se também aumento na taxa de mortalidade no grupo sobreviventes de UTI, o que levou a um intervalo livre de progressão menos favorável. Os grupos cuidados intensivos e hospitalizados diferiram significativamente em relação aos dados demográficos basais; no entanto, o intervalo livre de progressão permaneceu menos favorável em pacientes de UTI após o ajuste às variáveis demográficas disponíveis.

Esses achados, os primeiros a descreverem as taxas de progressão da doença após a UTI, têm consequências significativas nos 5% de pacientes com tumores sólidos que são admitidos na UTI.^(1)^ Com o tratamento anticâncer cada vez mais agressivo, dependemos da UTI como um facilitador das terapias oncológicas modernas.^(3)^ Embora devamos comemorar o aumento da sobrevida após a doença crítica, também é importante focar nos desfechos em longo prazo após a alta.

O Biobanco do Reino Unido proporcionou uma oportunidade única de avaliar os desfechos em longo prazo em uma grande população prospectiva. O Biobanco do Reino Unido teve uma taxa de resposta de apenas 5,5% e, consequentemente, é improvável que seja representativo da população em geral. Como acontece com muitas populações autosselecionadas, ocorreu um viés de seleção de voluntários saudáveis, e os entrevistados tinham uma incidência de câncer menor do que a população em geral. Os participantes tendem a pertencer a grupos socioeconômicos de maior renda e podem apresentar hábitos de saúde diferentes. As taxas relativamente baixas de câncer de pulmão observadas em nossas populações podem refletir esse viés de seleção. No entanto, ainda é possível generalizar os impactos da exposição e da doença na população, como o efeito da internação em UTI.^(9)^

O viés de sobrevida pode ocorrer quando os pacientes só podem ser incluídos em um estudo se forem submetidos a um processo de seleção. Neste estudo, os pacientes tiveram que sobreviver à UTI ou à hospitalização antes da inclusão. Pode haver diferenças nas características dos pacientes que sobreviveram à doença crítica em comparação com aqueles que morreram ou não tiveram doença crítica. Por exemplo, o câncer de pulmão está pouco representado em ambas as coortes, possivelmente devido às altas taxas de mortalidade observadas nesses pacientes.^(1)^ No entanto, como a exposição de interesse foi a UTI, acreditamos que os critérios de inclusão continuam sendo o método mais robusto para testar a hipótese.

Este é um estudo observacional e delimita associações e não causalidade. Embora a coorte da UTI possa ter uma taxa mais alta de doença metastática devido ao insulto sofrido durante a doença crítica, é provável que as coortes sejam diferentes de maneiras que não foram consideradas em nosso estudo. Nosso uso da análise multivariável tenta superar esses fatores de confusão quando os dados estão disponíveis; no entanto, pode haver diferenças não mensuradas entre as coortes que enfatizaram o efeito observado. A natureza retrospectiva deste estudo limitou os dados disponíveis para análise, e não foi possível fornecer uma descrição da doença crítica, das intervenções recebidas na UTI ou se esses fatores influenciaram os desfechos. A principal desvantagem do estudo é que nosso conjunto de dados não inclui dados contextuais acerca da tomada de decisão do médico sobre decisões de admissão, escalonamento do tratamento ou resposta ao tratamento de câncer de cada paciente.

## CONCLUSÃO

Este estudo revelou uma taxa maior de progressão da doença metastática em sobreviventes de doença crítica; no entanto, a causa exata dessa diferença não pode ser determinada, e não se explorou a repercussão de características específicas da doença crítica. Entretanto, as práticas de admissão em unidades de terapia intensiva não devem mudar a partir desses achados. Em vez disso, devemos considerar essa observação como geradora de hipóteses, com estudos futuros levando em conta a progressão da doença após a unidade de terapia intensiva, além da natureza e da gravidade da doença crítica, do grau de desregulação imunológica e do tratamento anticâncer subsequente.

## Data Availability

Os conjuntos de dados gerados e analisados durante este estudo estão disponíveis no Biobanco do Reino Unido, mas há restrições quanto à disponibilidade deles, pois foram usados sob licença neste estudo e não são públicos. No entanto, os dados estão à disposição por meio de solicitação ao Biobanco do Reino Unido.
